# Data for the spatiotemporal analysis of US global banks’ exposure to foreign counterparty risks

**DOI:** 10.1016/j.dib.2019.103964

**Published:** 2019-05-24

**Authors:** Ibrahim Niankara, Hassan Ismail Hassan

**Affiliations:** College of Business, Al Ain University of Science and Technology, Abu Dhabi, United Arab Emirates

**Keywords:** Foreign risks, Global banking, International banking, Risk management

## Abstract

This article Presents an extract of the fully consolidated data collected through the US Federal Financial Institutions Examination Council (FFIEC) reports, FFIEC 009 and FFIEC 009a [1]. The data is provided here as a Panel of Quarterly claims covering the 2017 fiscal year from last quarter 2016, to third quarter 2017. Following U.S. generally Accepted Accounting Principles (GAAP), it contains financial claims reported by 68 US banking organizations (including US holding companies owned by foreign banks, but excludes US branches of foreign banks), on foreign counterparties distributed across 71 countries in six world regions. From the original raw claims data we generate and include in this shared data, six accounting measures of foreign country risks (including Cross-border risk ratio, Foreign Office risk ratio, Derivative risk ratio, Ratio of Public Sector Claims, Ratio of Banking Sector Claims, and Ratio of non-bank financial sector claims) previously used to study the relative contribution of public sector, banking sector, and non-bank financial sector claims in U.S. global banks' exposure to foreign counterparties' default risks in [2]. The present article also presents a brief descriptive analysis of the various measures and their inter-relationships in characterizing US global banks exposures to foreign counterparties risks.

**Table:** Specifications table

Subject area	*Economics*
More specific subject area	*Financial Economics*
Type of data	*Tables, and Excel sheets*
How data was acquired	*Extracted From the 2017 Country Exposure Lending Survey (CELS), administered quarterly by the US Federal Financial Institutions Examination Council (FFIEC) using two reports, the FFIEC 009 and the FFIEC 009a. It aims to collect data on non-U.S. exposures of United States banks, savings associations, bank holding companies, Edge and/or Agreement corporations, and savings and loan holding companies (U.S. banking institutions) where claims on an ultimate-risk basis for a given country exceed one percent of the U.S. banking institution's total assets or 20 percent of its total capital, whichever is less.*
Data format	*Raw excel sheets along with R formatted data sets*
Experimental factors	*In addition to the raw claims data, Includes 6 computed risk ratios: Cross-border risk ratio, Foreign Office risk ratio, Derivative risk ratio, Ratio of Public Sector Claims, Ratio of Banking Sector Claims, and Ratio of non-bank financial sector claims.*
Experimental features	*Panel of quarterly compiled financial claims data on 71 foreign counterparties of US global banks, observed over four consecutive quarters starting from last quarter 2016, and ending with the 3rd quarter of 2017.*
Data source location	*71 counterparties distributed Worldwide across 6 regions: Africa, Asia Pacific, Eastern Europe, G10 and Luxembourg, Latin America, and Non-G10 Developed Countries.*
Data accessibility	*The Data is with this article*
Related research article	Niankara, I.; Ismail, H. (2018) ‘Relative Contribution of Public Sector, Banking Sector, and Non-Bank Financial Sector Claims in U.S. Global Banks' Exposure to Foreign Counterparties' Default Risks’*. Preprints (*https://doi.org/10.20944/preprints201803.0113.v1*)**[2]*

**Table:** 

**Value of the data** • This data article is intended to provide the academic and scientific community a better picture of the usability of the US country lending Survey (CLS), for financial risk analysis. • It provides a unique opportunity to analyze US financial institutions' exposure to foreign borrowers' risk of defaulting, and thereby allows for a better assessment, and coping of US financial system vulnerability sources. • This data provides an initial in-depth presentation of a treated version of the CLS data, which might inspire prospective investigations combining multi-year versions of the data, along with country level macro-economic indicators of risks, for various interesting country risks analyses.

## 1 Data

The data presented in this article is extracted from the 2017 Country Exposure Lending Survey (CELS), collected by the US Federal Financial Institutions Examination Council (FFIEC) using two reports, the FFIEC 009 and the FFIEC 009a [1]. The CELS provides quarterly data on non-U.S. exposures of United States banking institutions, where claims on an ultimate-risk basis for a given country exceed one percent of the U.S. banks' total assets or 20% of its total capital, whichever is less. The FFIEC 009 report collects detailed information on the distribution, by country, of claims on foreigners held by certain U.S. banks, savings associations, bank holding companies, savings and loan holding companies, and intermediate holding companies. While the FFIEC 009a is a supplement to the FFIEC 009 and provides specific information about the reporting institutions' exposures in particular countries.^1^

The report was initiated in 1977 as the FR 2036 report and was used to collect data on the distribution, by country, of claims on foreigners held by U.S. banks and bank holding companies. The Federal Deposit Insurance Corporation (FDIC)^2^ and the Office of the Comptroller of the Currency (OCC)^3^ collected similar information from institutions under their supervision. In March 1984, the FR 2036 became a Federal Financial Institutions Examination Council (FFIEC) report and was renumbered FFIEC 009. It was revised in March 1986 to provide more detail on guaranteed claims. In 1995, the report was revised to add an item for revaluation gains on off-balance-sheet items and an item for securities held in trading accounts, and several items were combined.

All data collected through the FFIEC 009 report are fully consolidated following U.S. generally accepted accounting principles (GAAP) and cover 68 US banking organizations (including US holding companies owned by foreign banks, but excludes US branches of foreign banks). All positions are reported on a gross basis, as of the last day of the quarter and in U.S. dollars regardless of the currencies in which the balances are denominated^4^

The key descriptive features of the data are provided in Table 1, Table 2 and Fig. 1, Fig. 2, Fig. 3, Fig. 4, Fig. 5, Fig. 6, Fig. 7, Fig. 8, Fig. 9, Fig. 10 below. All graphical and numerical descriptive statistics were produced using the R statistical software [3]. (See attached computer R codes for more details).

**Table 1:** Descriptive Statistics (mean and standard deviation in parenthesis) for the risk ratios.

Variables (%)	Q4-2016	Q1-2017	Q2-2017	Q3-2017	Total
CrxBordRR	56.73	58.35	57.73	58.44	57.81
	(28.79)	(28.46)	(28.69)	(28.84)	(28.56)
ForeignOfficRR	30.51	29.72	30.22	29.77	30.05
	(26.16)	(25.57)	(26.03)	(25.97)	(25.81)
DerivativRR	4.97	4.14	4.26	3.99	4.34
	(6.66)	(5.08)	(5.08)	(4.76)	(5.43)
PubSectRR	30.58	31.17	32.16	32.17	31.52
	(20.88)	(20.56)	(21.71)	(21.77)	(21.14)
BankSectorRR	21.22	20.72	20.25	19.88	20.52
	(18.16)	(17.29)	(17.23)	(17.07)	(17.36)
NonBkFinSectRR	12.80	11.76	12.01	12.23	12.20
	(17.20)	(16.48)	(16.89)	(17.15)	(16.85)

**Table 2:** Correlation matrix, along with the 95% confidence intervals on the six ratios of foreign risks.

	CrxBordRR	ForeignOfficRR	DerivativRR	PubSectRR	BankSectorRR	NonBkFinSectRR
CrxBordRR	1	−0.54 (−0.62, −0.46)	0.23 (0.12, 0.33)	0.09 (−0.02, 0.20)	0.35 (0.25, 0.44)	0.30 (0.20, 0.40)
ForeignOfficRR		1	−0.22 (−0.33, −0.12)	0.38 (0.28, 0.47)	−0.08 (−0.19, 0.04)	−0.18 (−0.29, −0.07)
DerivativRR			1	−0.14 (−0.25, −0.03)	0.24 (0.13, 0.34)	0.31 (0.21, 0.41)
PubSectRR				1	−0.23 (−0.34, −0.12)	−0.32 (−0.42, −0.22)
BankSectorRR					1	−0.10 (−0.21, 0.02)
NonBkFinSectRR						1

**Fig. 1:**
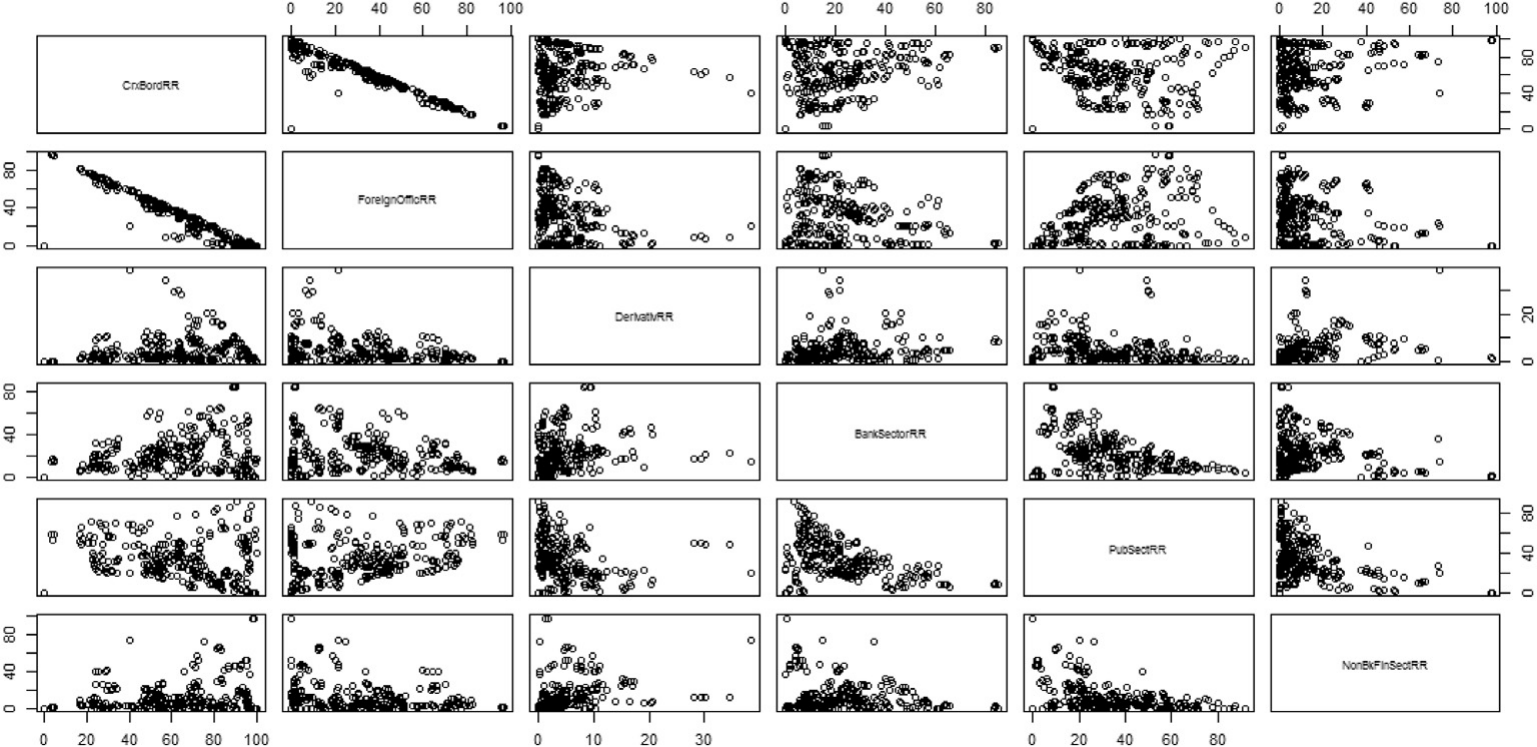
Pair-wise graphical correlation tests between the six ratios of foreign risks.

**Fig. 2:**
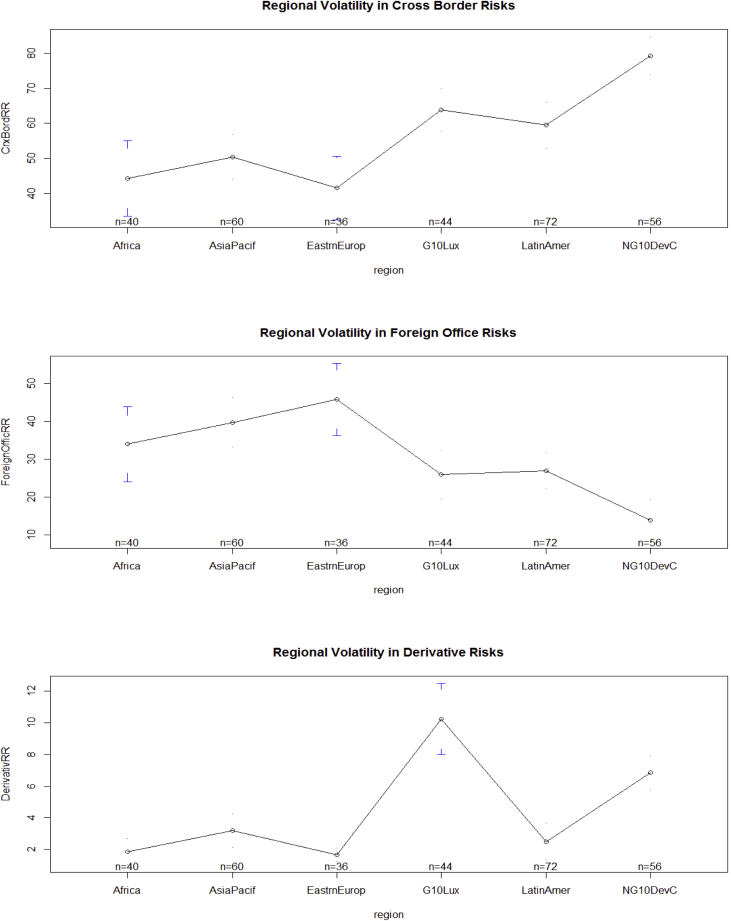
Aggregate regional volatility in cross-border risks (top panel), foreign office risks (middle panel), and derivative risks (lower panel).

**Fig. 3:**
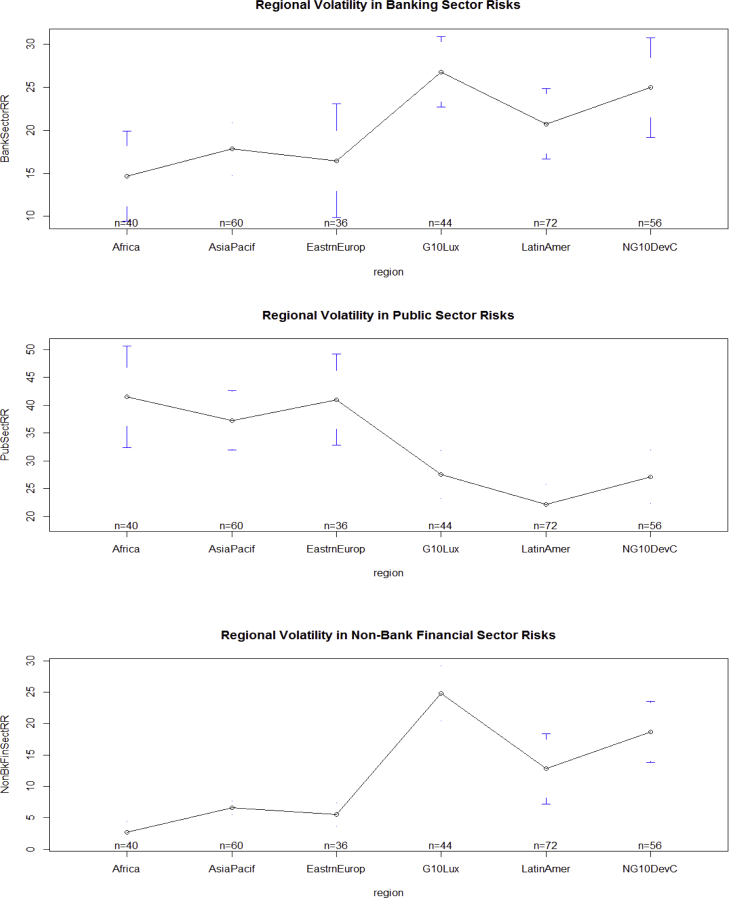
Aggregate regional volatility in public sector risks (top panel), banking sector risks (middle panel), and non-bank financial sector risks (lower panel).

**Fig. 4:**
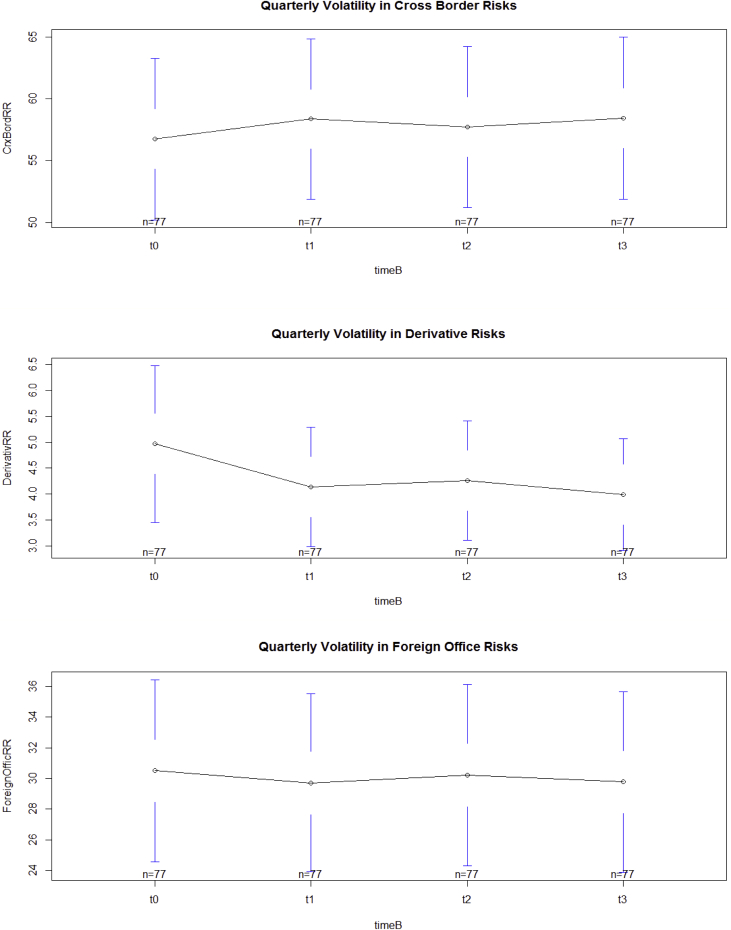
Aggregate quarterly volatility in cross-border risks (top panel), foreign office risks (middle panel), and derivative risks (lower panel).

**Fig. 5:**
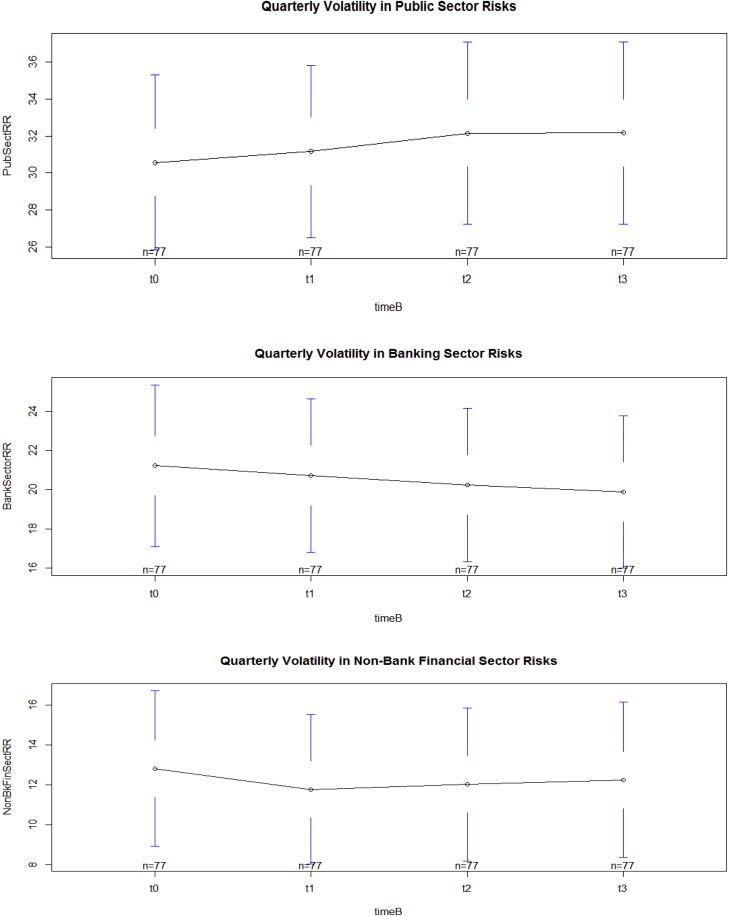
Aggregate quarterly volatility in public sector risks (top panel), banking sector risks (middle panel), and non-bank financial sector risks (lower panel).

**Fig. 6:**
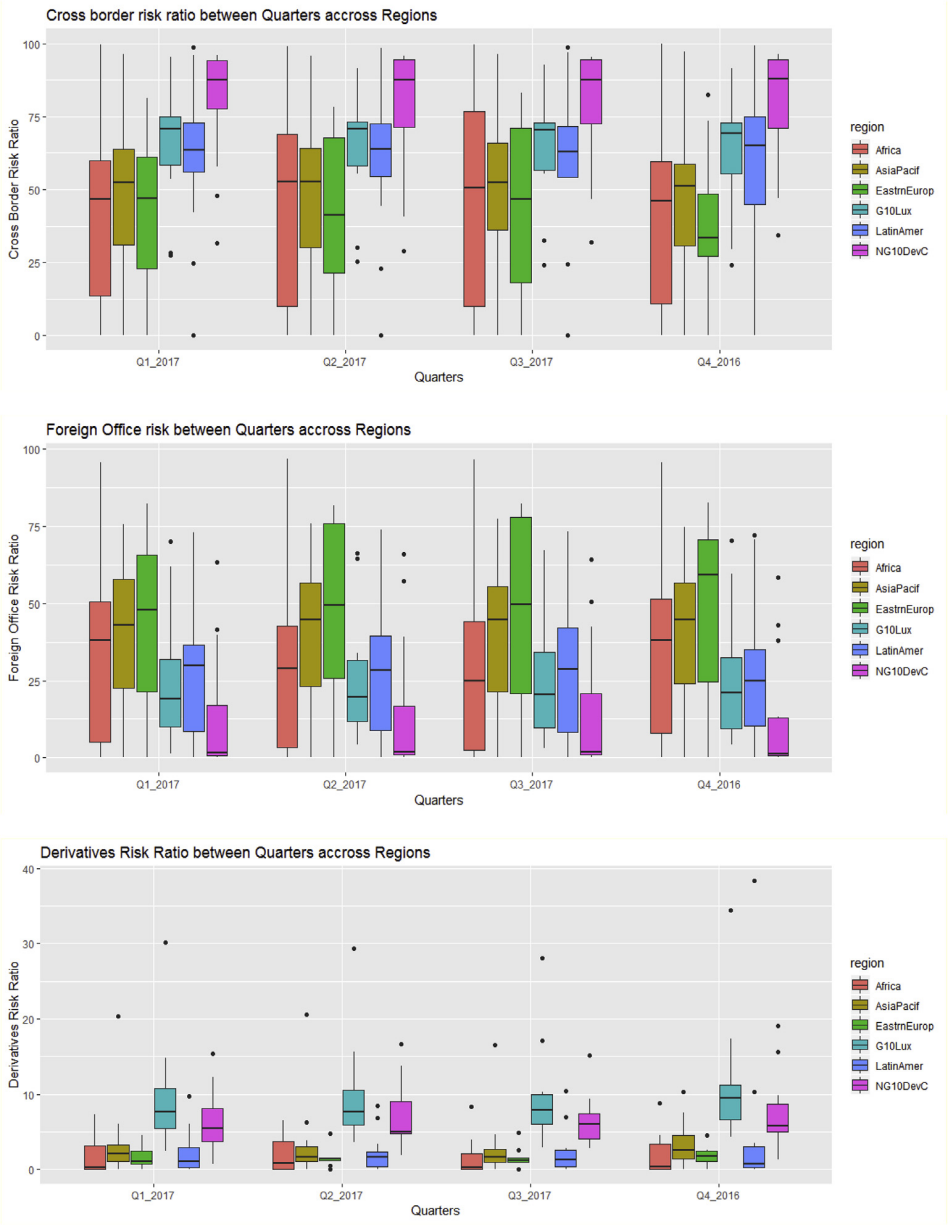
Boxplots of the cross-border risk ratio (top panel), foreign office risk-ratio (middle panel), and derivative risk ratio (bottom panel) between quarters across regions.

**Fig. 7:**
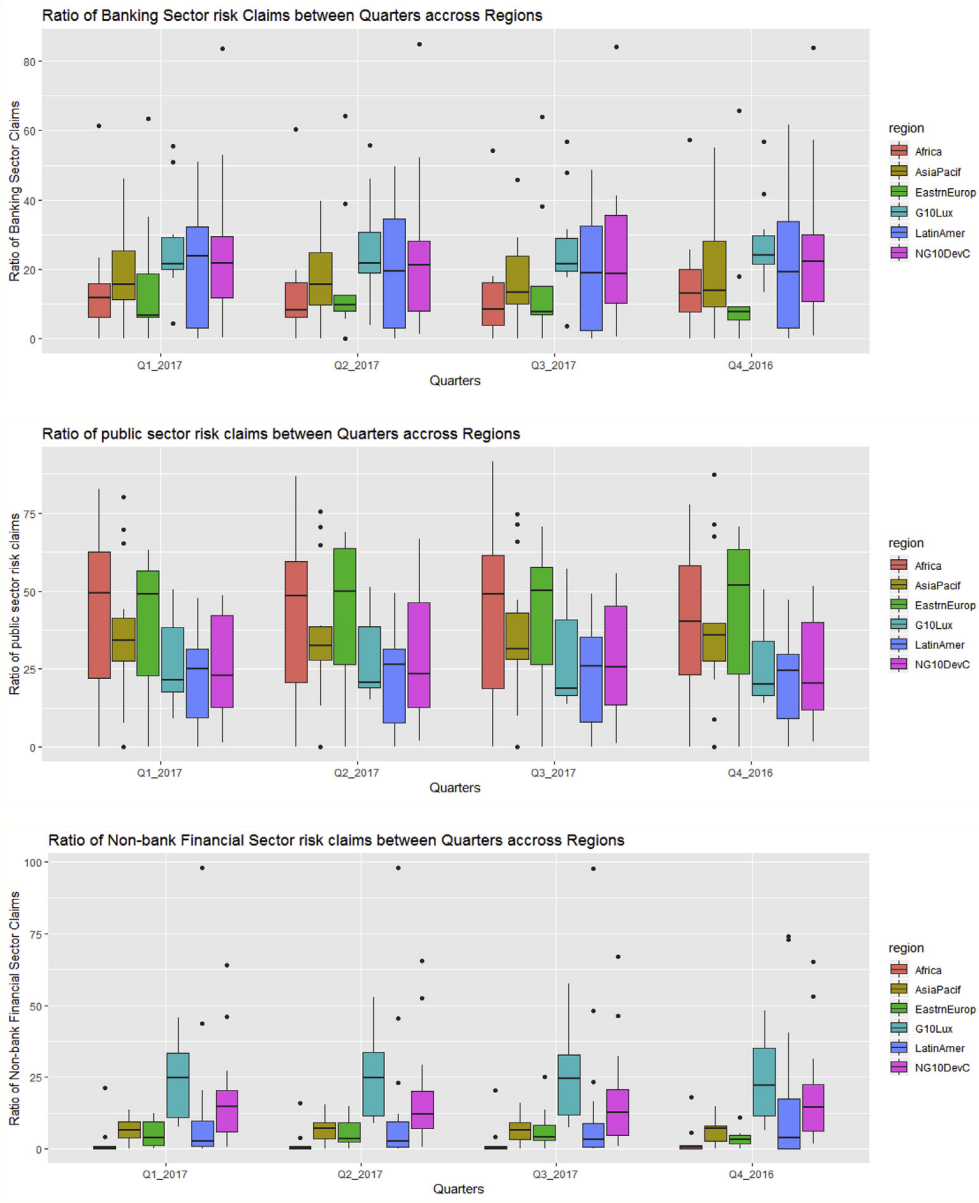
Boxplots of the ratio of banking sector claims (top panel), ratio of public sector claims (middle panel), and the ratio of non-bank financial sector claims (bottom panel) between quarters across regions.

**Fig. 8:**
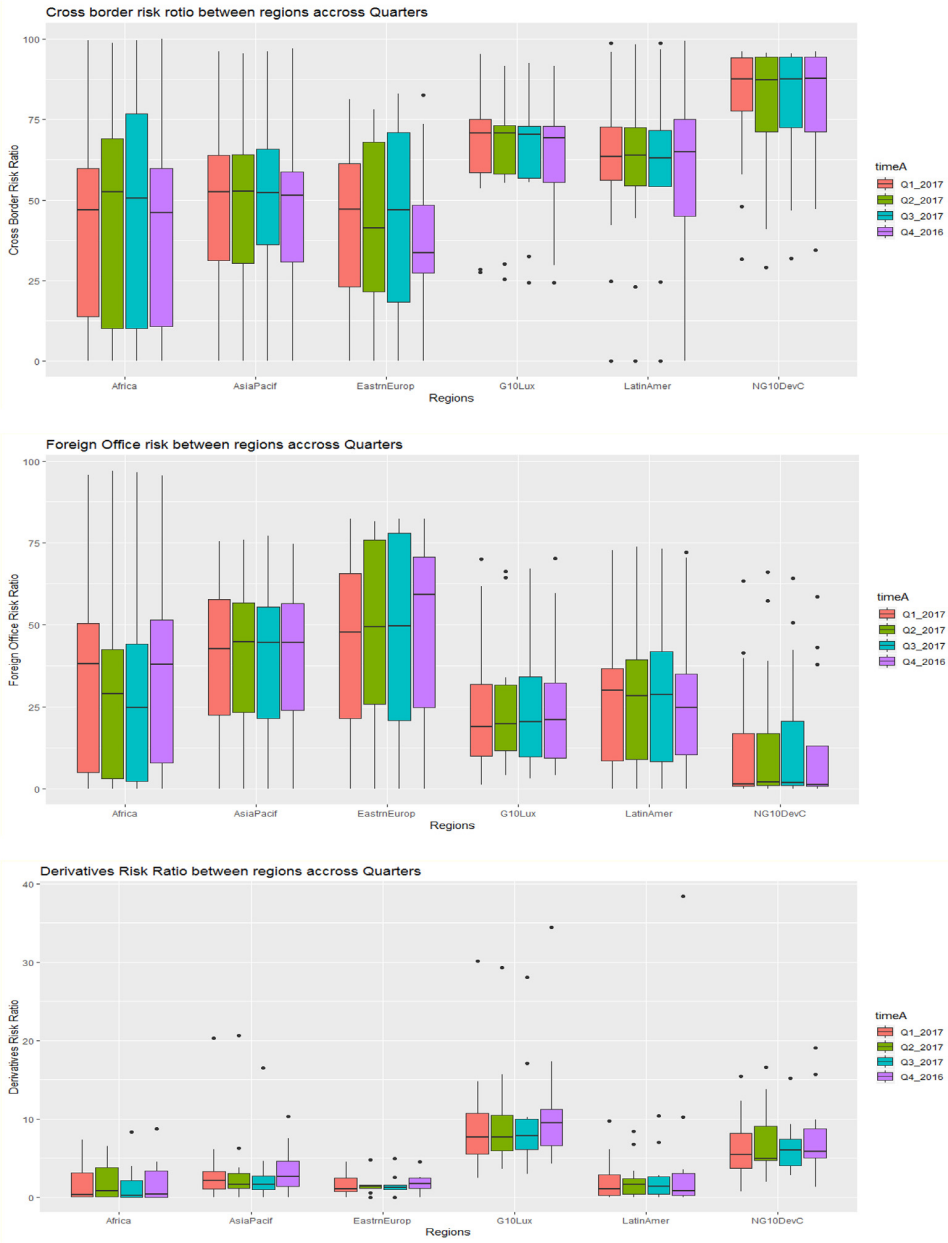
Boxplots of the cross-border risk ratio (top panel), foreign office risk-ratio (middle panel), and derivative risk ratio (bottom panel) between regions across quarters.

**Fig. 9:**
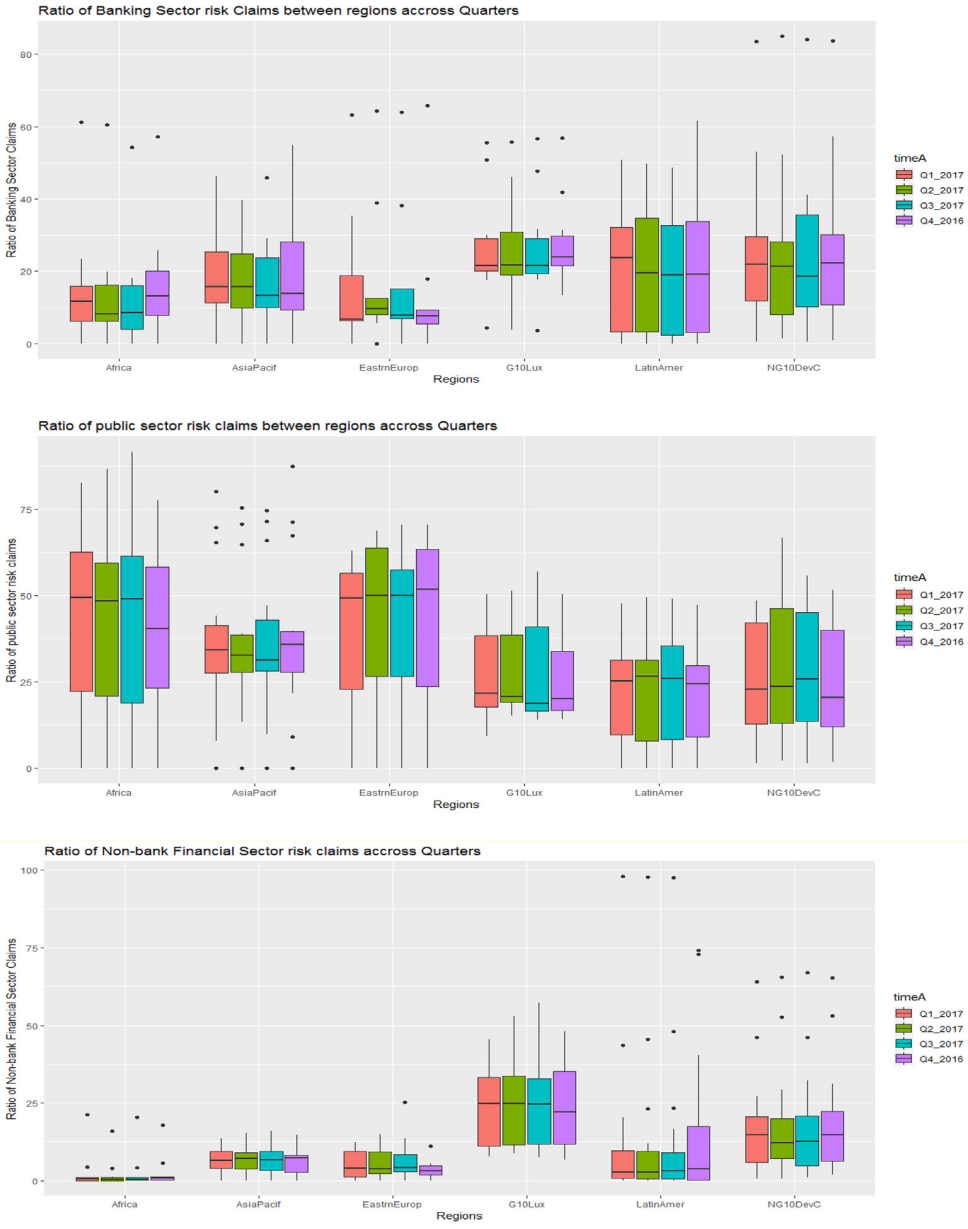
Boxplots of the ratio of banking sector claims (top panel), ratio of public sector claims (middle panel), and the ratio of non-bank financial sector claims (bottom panel) between regions across quarters.

**Fig. 10:**
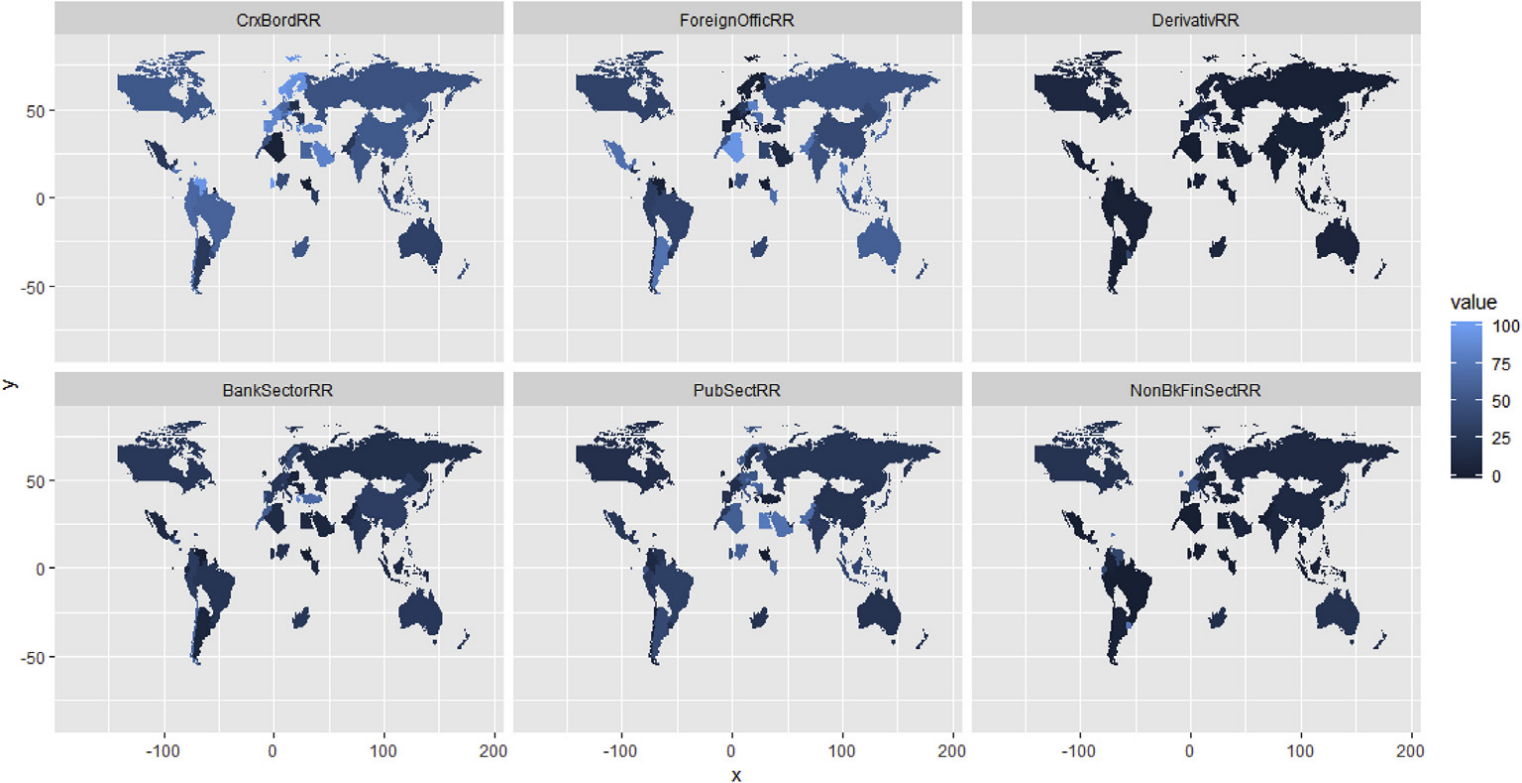
Global Mapping of the six ratios of U.S. global banks' foreign risk exposure. Source: Author's construction using the computed risk ratios from the shared data, in combination with the world geospatial information (Longitudinal and Latitudinal coordinates) provided in the R library “mapdata” [4].

## 2 Experimental design, materials, and methods

In order to describe US banking organizations' sensitivity to global risk exposure, we compile the raw claims data into a panel of quarterly data on 71 countries observed over four quarters starting from the last quarter of 2016, and ending with the 3rd quarter of 2017. We then derive ratios of foreign risk, using the cross-border risk claims, the foreign-office risk claims, and the claims from derivative products on an ultimate-risks basis.^5^ Following the standard in the international banking system, we rely on country risk claims to measure the exposure of reporting U.S. banks to an event that might severely limit the ability of borrowers in a foreign country to repay their debt.^6^ Since total Country risk claims are the sum of cross-border claims, foreign-office claims on local residents and claims from derivative products on an ultimate-risk basis [1], we derive our six foreign risk ratios as:

### 2.1 Cross-border risk ratio (CrxBordRR)

A measure of the share of total risk exposure attributable to cross-border risks (which describe the volatility of returns on international investments caused by events associated with a particular country, as opposed to events associated solely with a particular economic or financial agent). It is computed as:

$$CrxBordRR=\frac{\;\;Cross\;Border\;Claims}{Total\;Country\;Risk\;Claims}*100$$

### 2.2 Foreign office risk ratio (ForeignOfficRR)

A measure of the share of total risk exposure that is attributable to a global bank's risk claims on local residents of its foreign offices. It is computed as:

$$ForeignOfficRR=\frac{\;\;Foreign\;Office\;Claims}{Total\;Country\;Risk\;Claims}*100$$

### 2.3 Derivative risk ratio (DerivativRR)

A measure of the share of total risk exposure that is attributable to a global bank's derivative claims on foreign counterparties, on an ultimate-risk basis. It is computed using the following formula:

$$DerivativRR=\frac{Derivative\;Product\;Claims\;\;}{Total\;Country\;Risk\;Claims}*100$$

### 2.4 Ratio of public sector claims (PbSctRR)

A measure of the share of total risk exposure that is attributable to a global bank's risk claims on foreign governments (or foreign public sector). It is computed as:

$$PbSctRR=\frac{\;\;Total\;Ultimate\;Claims\;on\;Foreign\;Public\;Sector}{Total\;Country\;Risk\;Claims}*100$$

Both, the ratio of banking sector claims (BkSctRR) and the ratio of non-bank financial sector claims (NBkFinSctRR) are measures of relative risk exposures to foreign private sector. However, the distinction between banking sector, and non-bank financial sector provides another level of detail into global banks’ ability to trace the root cause of risk exposure, for a more targeted foreign risk management strategy, when and where needed. The measures are computed as shown below:

### 2.5 Ratio of banking sector claims (BkSctRR)

$$BkSctRR=\frac{\;\;Total\;Ultimate\;Claims\;on\;Foreign\;Banking\;Sector}{Total\;Country\;Risk\;Claims}*100$$

### 2.6 Ratio of non-bank financial sector claims (NBkFinSctRR)

$$NBkFinSctRR=\frac{Total\;Ultimate\;Claims\;on\;Foreign\;\;Nonbank\;financial\;sector\;\;}{Total\;Country\;Risk\;Claims}*100$$

Figure (12) below provides a spatial representation of the global distribution of these six risk measures (ratios). Note that the darker the color for a given country, the lower the level of risk exposure from counterparties in that country, while the lighter the color the higher the risk exposure from that country. Due to aggregations in the original raw CELS data, this map only represents all the countries explicitly named in the dataset, but excludes those falling under the umbrella of “others” such as: “other non-G10 Developing countries”, “Other Eastern European Countries”, “Other Latin American and Caribbean countries” “Other Asian and Pacific Island Countries”, and “Other African Countries”.

## Footnotes

1 Total cross-border exposures reported on the FFIEC 009a align with the cross-border disclosures reported in the U.S. Security and Exchange Commission's (SEC) Form 10-K. However, cross-border exposures differ from country risk exposures and the differences are outlined in U.S. banking institutions' SEC Form 10-K.

2 Represents an independent agency created by the U.S. congress to maintain stability and public confidence in the nation's financial system by insuring deposits, examining and supervising financial institutions for safety and soundness and consumer protection. See official page at https://www.fdic.gov.

3 an Independent bureau of the U.S. Department of the Treasury with the mission to ensure that national banks and federal savings associations operate in a safe and sound manner, provide fair access to financial services, treat customers fairly, and comply with applicable laws and regulations. See official page at https://www.occ.treas.gov.

4 With the following exception: (i) Trading assets and trading liabilities in the same exact security may be reported on a net basis, also referred to as ``CUSIP netting.'' (ii) Positive fair values of derivative contracts may be offset against negative fair values if, and only if, the transactions were executed with the same counter-party under a legally enforceable master netting agreement.

5 That is, by country of guarantor after applying adjustments for guarantees or eligible collateral. Eligible collateral is collateral that is (a) liquid and readily realizable, and (b) realizable outside of the country of residence of the borrower. Eligible collateral includes cash and investment grade debt or marketable equity securities.

6 The risk of non-compliance on claims in an ultimate-risk basis for a given country exceeding 1% of the reporting banks' total assets or 20% of its total capital (whichever is less) is an accepted risk measure within the context of international banking (see the E16 Endnotes (1 and 4), at the bottom of the FFIEC 009 report in PDF format) [1].
